# Whole-Genome Profiles of Malay Colorectal Cancer Patients with Intact MMR Proteins

**DOI:** 10.3390/genes12091448

**Published:** 2021-09-20

**Authors:** Wan Khairunnisa Wan Juhari, Khairul Bariah Ahmad Amin Noordin, Andee Dzulkarnaen Zakaria, Wan Faiziah Wan Abdul Rahman, Wan Muhamad Mokhzani Wan Muhamad Mokhter, Muhammad Radzi Abu Hassan, Ahmad Shanwani Mohammed Sidek, Bin Alwi Zilfalil

**Affiliations:** 1 Human Genome Centre, School of Medical Sciences, Universiti Sains Malaysia, Kubang Kerian 16150, Kelantan, Malaysia; khairunnisa.juhari@gmail.com; 2 Malaysian Node of the Human Variome Project, School of Medical Sciences, Universiti Sains Malaysia, Kubang Kerian 16150, Kelantan, Malaysia; 3 School of Dental Sciences, Universiti Sains Malaysia, Kubang Kerian 16150, Kelantan, Malaysia; kbariah@usm.my; 4 Department of Surgery, School of Medical Sciences, Universiti Sains Malaysia, Kubang Kerian 16150, Kelantan, Malaysia; andee@usm.my (A.D.Z.); mokhzani@usm.my (W.M.M.W.M.M.); 5 Department of Pathology, School of Medical Sciences, Universiti Sains Malaysia, Kubang Kerian 16150, Kelantan, Malaysia; wfaiziah@usm.my; 6 Clinical Research Centre, Hospital Sultanah Bahiyah, Alor Star 05460, Kedah, Malaysia; drradzi91@yahoo.co.uk; 7 Surgery Department, Hospital Raja Perempuan Zainab II, Kota Bharu 15200, Kelantan, Malaysia; drahmad69@gmail.com

**Keywords:** whole-genome sequencing, colorectal cancer, Malay, olfactory signalling pathway

## Abstract

Background: This study aimed to identify new genes associated with CRC in patients with normal mismatch repair (MMR) protein expression. Method: Whole-genome sequencing (WGS) was performed in seven early-age-onset Malay CRC patients. Potential germline genetic variants, including single-nucleotide variations and insertions and deletions (indels), were prioritized using functional and predictive algorithms. Results: An average of 3.2 million single-nucleotide variations (SNVs) and over 800 indels were identified. Three potential candidate variants in three genes—*IFNE, PTCH2* and *SEMA3D*—which were predicted to affect protein function, were identified in three Malay CRC patients. In addition, 19 candidate genes—*ANKDD1B, CENPM, CLDN5, MAGEB16, MAP3K14, MOB3C, MS4A12, MUC19, OR2L8, OR51Q1, OR51AR1, PDE4DIP, PKD1L3, PRIM2, PRM3, SEC22B, TPTE, USP29* and *ZNF117*—harbouring nonsense variants were prioritised. These genes are suggested to play a role in cancer predisposition and to be associated with cancer risk. Pathway enrichment analysis indicated significant enrichment in the olfactory signalling pathway. Conclusion: This study provides a new spectrum of insights into the potential genes, variants and pathways associated with CRC in Malay patients.

## 1. Introduction

Colorectal cancer (CRC) is one of the leading causes of cancer worldwide. It is the third most common cancer worldwide and the second most common cause of death [1]. Geographical and distribution differences influencing the incidence of CRC have been observed across the world, including an accelerated incidence rate in several Asian countries [2,3]. According to the Malaysia National Cancer Registry 2008–2013, CRC is one of the most common cancers in men and the third most common cancer in women [4].

Hereditary colorectal cancers are caused by highly penetrant mutations, such as those involved in tumour suppression or in the DNA mismatch repair system, including Hereditary Nonpolyposis Colorectal Cancer (HNPCC), Familial Adenomatous Polyposis (FAP), MYH-associated polyposis, and the rare hamartomatous polyposis syndromes [5]. Hereditary nonpolyposis colorectal cancer (HNPCC), also known as Lynch syndrome (LS), is an autosomal dominant cancer syndrome that is known to be the most common hereditary cancer, accounting for 5–10% of total CRC [6]. Individuals with LS are characterized by a high tendency to develop cancers in the extracolonic organs, such as endometrium, stomach, ovary, small bowel, hepatobiliary tract, renal pelvis, ureter, skin, and brain [7,8]. In the context of familial colorectal cancer, the genetic causes of familial adenomatous polyposis and LS have been well documented, with over 30% of all CRC cases having been identified to carry underlying genetic factors [5]. Mismatch repair (MMR) genes, including *MLH1*, *MSH2*, *MSH6*, and *PMS2*, are the most common genes that cause germline mutations in LS, with almost 90% of the cases diagnosed being associated with mutations in the *MLH1* and *MSH2* genes [9,10].

Individuals may develop a hereditary cancer syndrome when they acquire an inherited mutation, thus having an increased risk of developing certain tumours, which can appear at a relatively early age. In most known hereditary malignant syndromes, the increased risk is due to the mutation of a single gene, making these pathologies monogenic hereditary diseases. The affected genes commonly control the cell cycle or are involved in the process of repairing DNA damage. Non-hereditary tumours (sporadic cases) are also caused by an increased incidence of mutations in these genes; however, in sporadic cases, the genetic changes have newly developed in the cells of a tissue, causing somatic mutations, and are absent in other body cells [11].

In addition, there is a number of common low-risk loci identified in other studies which are known to contribute to an increased risk of both sporadic and hereditary cases of CRC [12,13]. With recent advancements in human genetic research, the technological progress in sequencing linked to next-generation sequencing (NGS) has led to an increase in knowledge and a better understanding of genetic mutations in cancer cells and pathway alterations, serving to create new models and enhance findings in the biology of cancer [14]. Whole-genome sequencing (WGS), which is a part of NGS, can be utilised to identify additional possible mutations and/or variants associated with CRC. The NGS technology through whole-genome sequencing has also revealed numerous single-nucleotide polymorphisms and somatic mutations in cancer genomes which had not been previously reported [15]. Although most inherited variants common in human populations have been discovered and are listed in databases, there are myriad rare inherited single-nucleotide polymorphisms (SNPs) and structural variants yet to be found and, in most cancer genomes, these rare germline mutations are present in higher frequency than somatic mutations [14]. Hence, the use of WGS has led to the discovery of causative mutations for specific types of cancer [16,17].

The discernible difference between whole-exome sequencing (WES) and whole-genome sequencing (WGS) is that WES can capture or identify variants only in genes’ coding regions, while WGS is more efficient in identifying variants in the entire genome [18]. WGS itself is able to accurately detect and identify a higher percentage of true positive single-nucleotide variants (SNVs) in the exome [19]. Therefore, in this study, WGS was performed to capture various types of genomic alterations, in order to discover and further determine high-impact variants and other mutations, including rare mutations in other genes, that may be associated with an increased risk of CRC, particularly, in our Malay patients who fulfilled the Bethesda criteria.

## 2. Materials and Methods

### 2.1. Selection of Patients

Ethical approval was obtained from the Research and Ethics Committee, Universiti Sains Malaysia (USMKK/PPP/JEPeM [259.3.(9)]), and the Medical Research and Ethics Committee (MREC), Ministry of Health (NMRR-12-856-11623). All patients were selected from three hospitals, i.e., the Hospital Universiti Sains Malaysia (USM) and two hospitals under the Ministry of Health of Malaysia: Hospital Raja Perempuan Zainab II, Kota Bharu, Kelantan, and Hospital Sultanah Bahiyah, Alor Setar, Kedah. Sample recruitment was only focused on the Malay probands, due to the demographic pattern and to the fact that the majority of patients in these three hospitals were Malays. In addition, there is a scarcity of data for HNPCC in Malaysia, including mutations and/or polymorphisms specifically for the Malay population—the biggest ethnic group in Malaysia. In Malaysia, a multi-ethnic country with three different major ethnic groups (Malay, Chinese, and Indian), at present there are limited data on HNPCC variants in the Chinese population, but none have been reported for the Malay and Indian populations. Therefore, we decided to include and focus on patients of Malay ethnicity only. The CRC patients who fulfilled at least one of the revised Bethesda Criteria were enrolled into this study, according to the inclusion and exclusion criteria (see Table 1). Informed consent was obtained for each patient prior to sample collection. Seven patients were enrolled into this study, with five of them being unrelated patients (denoted as F1, F2, F12, F18, and F19), and two from the same family (denoted as F5 and F8).

### 2.2. Immunohistochemical Screening

Immunohistochemical staining was performed on formalin-fixed, paraffin-embedded (FFPE) tissue from a biopsy or resected bowel specimen. Immunohistochemical staining using four types of MMR antibodies—MLH1, MSH2, MSH6, and PMS2—and semi-quantitative scoring assessment were performed, as described previously by our groups [20].

### 2.3. Whole-Genome Sequencing

Genomic DNA was extracted from blood by a QIAamp DNA Blood Kit, following the manufacturer’s protocol (Qiagen, Hilden, Germany). Library preparation was carried out using TruSeq Nano DNA HT (Illumina, San Diego, CA, USA) prior to library quantification. The DNA libraries were then clustered onto the HiSeqX flow cell and were sequenced using the HiSeqX platform of the Beijing Genome Institute (BGI). Base calling was processed by an Illumina pipeline with default parameters, and the sequences of each patient were generated as 150 base pair (bp) paired-end reads. The adapter sequences of unknown bases, low-quality reads, and reads with unknown bases corresponding to more than 10% were removed from the raw sequencing data, prior to sequence alignment to the reference genome. The filtered reads were aligned to a human genome reference (GRCh37/hg19) using the Burrows–Wheeler Aligner (BWA) (Supplementary Table S1) [21]. Duplicate reads caused by PCR were further marked by Picard tools prior to variant calling. SOAPsnp was used to call single-nucleotide variants (SNVs) [22], and small insertion/deletions (indels) were detected by Samtools [23]. Following sequencing, the predicted effects of each variant were annotated using Annovar [24]. A list of databases and additional prediction algorithms were used to estimate the allele frequencies of each variant, including dbSNP [25], 1000 Genomes Project [26], and 1000 Genomes East Asian Project [27]. The variant functional effects and pathogenicity were further predicted by Polymorphism Phenotyping v2 (PolyPhen2) [28], Sorting Intolerant From Tolerant (SIFT) [29], MutationAssessor [30] and Functional Analysis through Hidden Markov Models (FATHMM). Additional annotation to facilitate the characterization and interpretation of variants was carried out using a cancer-related database, the Catalogue of Somatic Mutations in Cancer (COSMIC) database [31]; a disease-related database, ClinVar [32]; the Human Gene Mutation Database (HGMD) [33]; a systematic review of the literature.

### 2.4. Variant Prioritization

In the present study, the variants were filtered to prioritize the causative variants to LS predisposition. For single-nucleotide variants (SNVs), variants in the coding region and high-impact variants were selected. The high-impact variants were identified to be functional variants, i.e., missense, nonsense, splice acceptor and splice donor variants [34]. Over 200 high-impact variants were identified in each patient, with an average of 76 high-impact variants classified as nonsense mutations. In addition, to fully characterize the plausible variants in our patients, the identification of rare SNVs was further carried out on the basis of the following criteria: synonymous and SNVs present in the coding region were excluded, as well as variants with no subsequent effect on amino acids. The SNVs present in the dbSNP141 (SNP database) and 1000 Genomes were also excluded, and we selected only variants that could actually be damaging and have an effect on protein function, through in silico prediction using SIFT and Polyphen2.

For prioritization of indels, only indels in coding regions were selected, including indels leading to disruptive in-frame insertion, disruptive in-frame deletions frameshift variants, frameshift mutations causing stoploss mutation, in-frame insertion, in-frame deletion, splice acceptor variants, splice region variants, and in-frame deletion causing stoploss mutation. Synonymous and indels that lay in non-coding regions, including indels with no functional protein annotation mapped to SIFT and PolyPhen2, were excluded for downstream interpretation. Rare indels, which were hypothetically considered as rare indels when those variants were not identified in dbSNP141 (SNP database) and 1000 Genomes database, were included in this study. The variants presented in the COSMIC database were included in order to ascertain the impact of the variants on human cancers.

Pathway enrichment analysis, using STRING (https://string-db.org/ (accessed on 26 August 2021)) [35] and Reactome (https://reactome.org/ (accessed on 28 August 2021)) [36], was performed to prioritize the non-synonymous variants harbouring nonsense single-nucleotide variants in the respective candidate genes. Interaction and correlation based on the knowledge-based pathway map was employed by String, based on Gene Ontology (http://www.geneontology.org/GO (accessed on 28 August 2021)) and KEGG (http://www.genome.jp/kegg/ (accessed on 28 August 2021)).

## 3. Results

### 3.1. Variant Identification in Patients with Intact MMR Protein Expression

Immunohistochemical staining of the seven studied patients showed no loss of expression in the four MMR antibodies MLH1, MSH2, MSH6 and PMS2. The resulting variants in MMR genes were mostly discovered in intronic regions, suggesting that the variants may have no effect on protein expression. In the present study, an average of 3.2 million single nucleotide variations were identified in each genome, when mapping against the human genome reference sequence assembly GRCh37, also known as hg19 (Supplementary Table S2). The genome sequences covered over 99% of the reference sequence, in an approximate range of 44- to 52-fold sequencing depth for each sample. The whole-genome sample data of the studied patients were filtered and prioritized to fully characterize the high- and low-risk loci that may be associated with CRC in Malay patients fulfilling the Bethesda criteria. We discovered a non-synonymous polymorphism in exon 3 of the *EPCAM* gene c.344T>C (p.Met115Thr) (rs1126497) in the seven studied patients.

In addition, three female patients were identified to harbour a nonsense variant in the *IFNE* gene, c.211C>T (p.Gln71*) (rs2039381). Regarding this variant, in silico prediction using SIFT found that the variant was predicted to abolish protein function. Among the three patients with the variant in the *IFNE* gene, two were first-degree relatives (F5 and F8). Two rare heterozygous missense mutations—c.1307C>T (p.Ala436Val) in exon 10 of *PTCH2* and a mutation in the *SEMA3D* gene, c.278T>A (p.Leu93His) located at exon 2—were also exclusively identified in these patients. F5 and F8 were diagnosed with colorectal cancer at the age of 43 and 56 years, respectively. In silico prediction to further assess the functional consequences of these mutations was performed using SIFT, Poyphen2, MutationAssessor, RadialSVM, and FATHMM. Both SIFT and Polyphen2 predicted that these mutations would be deleterious, with scores of 0 and 0.8–0.9, respectively. For the mutation in the *PTCH2* gene, MutationAssessor and RadialSVM resulted in scores of 2.515 (Medium) and 1.0647 (Damaging), respectively, and the mutation in the *SEMA3D* gene resulted in scores of 3.765 (High) and 0.0534 (Damaging), respectively. Based on FATHMM prediction, both mutations were predicted to be pathogenic.

The SNVs were classified into high-, moderate-, and low-impact, based on the annotation algorithms. SNVs that lead to protein truncation could have a highly disruptive effect on gene function, whereas SNVs that influence only protein effectiveness are most likely to have only a moderate effect, and synonymous SNVs that are unlikely to change protein behaviour probably have a minimal effect. In these studied patients, a total of more than 200 SNVs that could affect gene function (high impact) were identified, and those SNVs that caused stop-gain mutations (nonsense mutations) were selected for further pathway enrichment analysis. In patient F1, a total of 81 nonsense mutations were identified from 274 high-impact SNVs, whereas, in patient F2, 73 nonsense mutations were identified out of 265 high-impact SNVs. For patient F5, 82 nonsense mutations were discovered from 286 high-impact SNVs identified, while a total of 73 nonsense mutations were identified in patient F8 from 274 high-impact SNVs. Of 270 high-impact SNVs found in patient F12, 78 SNVs were identified to be nonsense mutations. A total of 73 nonsense mutations were identified in patients F18 and F19, from 262 and 267 high-impact SNVs, respectively. Twenty nonsense mutations in 19 genes were prioritized, taking into account only the shared mutations among these seven CRC patients (Table 2). We also identified 15 nonsense and 7 indels occurring in five or six samples. Two variants within these five or six samples were identified in two genes, *KRT10* (p.Gly490_Gly493del/c.1468_1479delGGCCACGGCGGC) and *MTSS1* (c.1417-37delT).

### 3.2. Pathway Analysis

Pathway enrichment analysis was then performed for all the candidate genes harbouring nonsense mutations. For this, Reactome, a curated database of pathways and reactions in human biology, was used. From the test, a probability score was produced, which was then corrected for false discovery rate (FDR) using the Benjamani–Hochberg method. Based on our submitted data, the olfactory signalling pathway was discovered to be the most significant pathway for each patient (Supplementary Table S3). In concordance, Gene Ontology (GO) enrichment analysis and KEGG pathway, performed using the String tool, revealed that the molecular function and pathway identified were primarily related to olfactory receptor activity and olfactory transduction pathway, respectively (Table 3). In addition to SNVs, over 800,000 insertions and deletions (indels) were identified in each patient. Eight indels of two in-frame insertions, two frameshift deletions, three frameshift insertions, and one disruptive in-frame deletion were considered as rare indels in the Malay CRC candidate genes *CDK11B*, *CCDC144NL*, *GOLGA8R*. *MAFA*, *MUC6*, and *PRIM2* (Table 4). Pathway enrichment analysis was also performed in all candidate genes harbouring rare indels in these seven patients. Several significant pathways were identified from the submitted data, including a pathway that caused colorectal cancer by defective GALNT12.

## 4. Discussion

Whole-genome sequencing (WGS) was employed to further discover the molecular basis of predisposition to CRC in patients fulfilling the Bethesda criteria, which demonstrated an intact protein expression of four common MMR genes: *MLH1*, *MSH2*, *MSH6* and *PMS2*. We hypothesized that other genes could contribute to the CRC predisposition and sought to further identify other pathways that may be associated with the candidate genes. In this study, WGS was employed over exon capture approaches, in order to fully discover whether the causal variants would reside in known coding regions or other non-coding regions, as exome sequencing or other targeted approaches are only tailored to capture limited regions of variants [37]. Approximately 11% of the variants discovered by WGS have been reported to be missed by WES [38], and, even though WGS may fail to identify WES-specific variants, the number of variants missed by WGS has been found to be less significant [39]. Due to the massive amount of NGS data, a constructive approach must be considered to thoroughly select, filter and extract the functional variants from the data. Potential variants should be prioritized, including functional variants of uncommon polymorphisms, in order to search for likely true candidates for the studied disease [40]. The region-based annotation that has been implemented by ANNOVAR to classify the variants into specific genomic regions, such as intronic, intergenic, exonic, untranslated, splicing and non-coding RNA, including downstream and upstream genomic regions as well as their classes, such as synonymous, missense and frameshift variants [41], provides a useful handling step, as gene-based annotations cannot fully predict the functional consequences of variants outside protein coding regions [24].

A non-synonymous polymorphism, c.344T>C (p.Met115Thr) (rs1126497), in exon 3 of the *EPCAM* gene was discovered in the seven considered patients. This non-synonymous polymorphism has been previously reported as having a significant association with an increased risk of developing breast cancer in a Chinese population [42]. The location of this non-synonymous polymorphism in the thyroglobulin (TY) domain of the *EPCAM* gene suggests its role in inhibiting cathepsins, a family of cysteine proteases that are frequently secreted by tumour cells during metastasis [43]. The amino acid change of methionine to threonine in this TY domain may suggest its role in *EPCAM* gene function [42]. In addition, the association between *EPCAM* and *MSH2* was due to the simultaneous loss of EPCAM and MSH2 protein expression in colorectal cancer cases in *EPCAM* deletion carriers [44]. MSH2 inactivation was predicted in several patients with no MMR germline mutations but, in the presence of heterozygous germline deletion at the polyadenylation site in the exon 8 and 9 of *EPCAM* gene [45], the deletions were shown to cause a transcriptional read-through that silenced and inactivated the promoter of the *MSH2* gene located downstream of EPCAM gene [46]. Germline deletions in the last exon of the *EPCAM* gene may silence its neighbouring gene, *MSH2*, which is located 17 kb downstream of *EPCAM*, via promoter hypermethylation [47]. The germline deletion that causes *MSH2* inactivation was considered a novel mutation predisposing to HNPCC [48,49].

We discovered that the high-impact variants in this cohort of patients harboured nonsense variants, and these variants should be of primary interest in disease-related studies due to their potentially high cellular impact, as they include exonic missense, nonsense, stop-loss, frameshift, and splice site variants, which potentially affect protein function [41]. Nineteen candidate genes harbouring nonsense variants that may be involved in CRC in these Malay patients—*ANKDD1B*, *CENPM*, *CLDN5*, *MAGEB16*, *MAP3K14*, *MOB3C*, *MS4A12*, *MUC19*, *OR2L8*, *OR51Q1*, *OR51AR1*, *PDE4DIP*, *PKD1L3*, *PRIM2*, *PRM3*, *SEC22B*, *TPTE*, *USP29* and *ZNF117*—were further ascertained by consulting the literature and public databases for their possible clinical implications with respect to predisposition to other cancers. Claudin-5 is primarily expressed by the vascular endothelium and functions in the blood–brain barrier and pulmonary endothelial barrier, in addition to serving as a regulator of epithelial function [50]. Two different entries for the human claudin-5 protein with gene products of different size were found in the NCBI and Uniprot databases, which were 218 and 303 amino acids in length, respectively [51]. Considering the different types of gene products, a study was carried out to explore the coding sequence of *CLDN5*, and a reported SNP of rs885985 was found in the general population [51,52], as well as in our studied patients. The *CLDN5* allele introduces a stop codon and results in a claudin-5 open reading frame (ORF) with 218 amino acids. The presence of the G allele may introduce an overlapping ORF, which consequently encodes the two types of gene products. Immunoblotting of human lung tissue was, then, carried out to measure the size of the produced protein, which resulted in to be only 218-amino acid long [51]. A previous study discovered the role of MAP3K14 in the suppression of epithelial cell proliferation and its involvement in non-canonical NF-κB signalling during CRC development [53]. NF-κB-inducing kinase (NIK, also known as MAP3K14) signalling has been found to be essential in modulating the activity of the NF-κB pathway in pancreatic cancer [54], as the increased activity of the NF-κB pathway in pancreatic cancer caused cell proliferation and tumour development [55,56]. In addition, it has been reported that MS4A14 protein expression was detected in several cases of colorectal cancer, and retained expression was observed during the malignant transformation of tumours [57]. MS4A12 also functions in modulating EGFR signalling, the main tumour-promoting factor in colon cancer, causing tumour growth and survival, whereas loss of MS4A12 protein deteriorated EGFR-dependent cell functions [57]. Two other candidate genes harbouring indels were both identified to be associated with cancer predisposition. *MSS1* may be involved in the inhibition of CRC metastasis [58], and *KRT10* was reported to be highly expressed in hereditary skin cancer [59].

A majority of the identified variants should be validated and, in this case, biological knowledge including their molecular functions and interactions is essential to explore and confirm the role of these candidate variants and genes [41]. In addition, biological differences between mutations associated with human diseases and polymorphisms that cause stop codons should be further explored, as they may shed light into other molecular pathways at the basis of the disease [60]. Pathway enrichment analysis was further carried out by String and Reactome, using all genes of high-impact variants causing stop-gain mutations. This approach allowed us to identify genes enriched in several relevant biological and molecular processes. Given that the genes were selected based on their high-impact consequences on protein function, the olfactory signalling pathway was identified as the most significant pathway, based on the enriched candidate genes. Being the largest multi-gene family in the human genome, with approximately 3% of total human genes, the role of olfactory receptors (ORs) in cancer has been disregarded, due to their specific role in the olfactory epithelium [61]. Recent studies have also identified genes for potential ORs as alternative genes for the treatment of cancer, including *OR51E2*, which is involved in the regulation and proliferation of prostate cancer [62]. Another olfactory receptor (OR), OR51B4, has been observed to be highly expressed in the colon cancer cell line HCT116 [63]. Although, among the ORs enriched in the pathway analysis, OR51B4 was not discovered in our cohorts, our findings suggest that ORs could be potential genes for further exploration in colorectal cancer research.

While dealing with a vast number of candidate genes and variants, rare variants should be primarily considered for the prediction of functional effects [64]. In this study, synonymous variants and variants in non-coding regions were presumed to have non-functional impacts and were excluded [41], as well as variants that were less likely to be identified in the dbSNP. Among 16 genes harbouring rare missense mutations in the two first-degree relative patients, the best candidate genes and variants were then prioritized, based on in-silico prediction. Considering the detrimental effect and pathogenicity of these two mutations—c.1307C>T(p.Ala436Val) in the *PTCH2* gene and c.278T>A (p.Leu93His) in the *SEMA3D* gene—the functions of these genes were further delineated. A novel mutation in *PTCH2* was identified in an autosomal dominant disorder of naevoid basal cell carcinoma syndrome (NBCCS) in a Chinese population [65]. High *PTCH2* expression has also been observed in familial and sporadic basal cell carcinoma [66], and *PTCH2* is considered to be an important gene in murine medulloblastoma tumorigenesis [67]. However, the potential roles of this gene, specifically with regard to colorectal cancer predisposition, are yet unknown. However, the mutation c.1307C>T(p.Ala436Val) presented in these patients was predicted to block the protein function and may likely contribute to tumorigenesis. Several studies have determined the role of SEMA3D in the predisposition to several type of cancers, including breast cancer [68], glioma [69], and thyroid cancer [70]. Intriguingly, higher mRNA expression of *SEMA3D* mRNA has been observed in normal colorectal mucosa, as compared to the CRC tissues, suggesting that *SEMA3D* may function as a tumour suppressor gene in CRC progression [71]. *IFNE* has been identified as an apoptosis regulatory gene that can suppress cell proliferation in human colorectal cancer cells [72], despite its role in protecting the female reproductive tract against viral and bacterial infection [73].

Insertions and deletions are responsible for most genomic divergence, also in mammalian genomes [74]. Rare indels were identified in six genes—*CDK11B*, *CCDC144NL*, *GOLGA8R*, *MAFA*, *MUC6* and *PRIM2*—which harboured a total of eight rare functional indels. Two frameshift indels in *MUC6* caused a deletion (c.4712delC) and an insertion (c.4707_4708insA). In the normal colon, *MUC5AC* is rarely expressed, and there have been conflicting reports concerning the expression of *MUC6* in the colon [75]. However, *MUC6* expression has been reported to be associated with favourable outcomes in intermediate-stage (II and III) CRC patients [76]. Meanwhile, two frameshift indels were identified in the *CCDC144NL* gene. However, the gene function, with respect to predisposition towards cancer, remains unclear. Variations in the *CCDC144NL* gene were associated with poor prognosis and may facilitate cancer metastatic progression [77]. The *MAFA* gene, identified as harbouring a disruptive in-frame deletion in the seven considered patients, is known to be involved in oncogenic activities and cancer progression [78]. MAFA is one of the large Maf proteins that have been implicated in carcinogenesis, as demonstrated in cell culture, animal models, and cancer tissues [78]. Maf proteins have been identified to be involved in oncogenesis by the discovery of *v-maf* oncogene, which codes for the Maf protein member that causes fibrosarcoma in chickens [79]. *CDK11B*, characterized by an in-frame insertion in the proband, was known to likely be linked to predisposition to various human cancers [80]. *CDK11B* and its homologue gene encoding CDK11, a protein kinase that has been shown to be involved in the proliferation of various cancer cells, is involved in modulating the Wnt/β-catenin pathway in colon cancer [81]. The *PRIM2* gene, which is involved in synthesizing the Okazaki fragments in DNA replication, has been discovered as having the highest mutation rate in prostate cancer [82]. Among the several pathways enriched on the basis of the rare indels in the seven patients, defective GALTN12 signalling was identified as a significant pathway associated with colorectal cancer [83]. *GALNT12* was not identified in our cohorts; however, *MUC6*, a gene that encodes the mucin protein in epithelial tissue was enriched in this pathway. The GALNT family is classified as CAZy family GT27, and abnormality in one of the GALNT family genes, including *GALNT12*, may result in reduced glycosylation of mucins [84]. Mucin genes are mainly expressed in digestive organs such as stomach, small intestine and colon and may play a role in colorectal cancer [85].

## 5. Conclusions

This study provides new insight into the gene variants related to CRC predisposition in a Malay population. However, the small number of patients and family members recruited in this study resulted in a small number of samples available for the analysis; therefore, it was challenging to elucidate the role of the identified variants in the pathogenicity of CRC in our Malay cohorts. The analysis of whole-genome data allowed the discovery of a new spectrum of variants, including candidate genes, and pathways. It would thus be beneficial to verify these findings in a larger cohort of patients, so to further validate them, carry out a functional analysis and rule out variant segregation. The whole-genome sequencing approach used in this study has provided new molecular knowledge of CRC in the considered cohort of Malay patients.

## Figures and Tables

**Table 1:** Inclusion and exclusion criteria for the selection of the patients.

Inclusion Criteria
Malay patients (at least three generations and no admixture in the parental heritage) with colorectal cancer who fulfilled at least one of the following Bethesda Criteria: i. Colorectal Cancer (CRC) diagnosed in patient aged <50 years. ii. Presence of synchronous, metachronous colorectal, or other Lynch syndrome-related tumours *, regardless of age. iii. Patient with CRC and a first-degree relative with a Lynch syndrome-related tumour, with one of the cancers diagnosed at age <50 years. * Lynch syndrome-related tumours include colorectal, endometrial, stomach, ovarian, pancreas, ureter, renal pelvis, biliary tract and brain tumours, sebaceous gland adenomas and keratoacanthomas and carcinomas of the small bowel.
Exclusion Criteria
(1) Non-Malay (Chinese and Indian ethnic groups). (2) Patients with Familial Adenomatous Polyposis

**Table 2:** Nonsense mutations identified in the seven whole-genome samples.

Gene	Transcript	Codon Change	Chr	Start	End	Ref	Obs	Frequency *
***MOB3C***	NM_145279.4:p.Arg24*/c.70C>T	Cga/Tga	chr1	47080679	47080679	G	A	0.642173
***PDE4DIP***	NM_001198834.4:p.Trp2351*/c.7053G>A	tgG/tgA	chr1	144852390	144852390	C	T	-
***PDE4DIP***	NM_001198834.4:p.Arg622*/c.1864C>T	Cga/Tga	chr1	144915561	144915561	G	A	-
***OR2L8***	NM_001001963.1:p.Tyr289*/c.867T>A	taT/taA	chr1	248113026	248113026	T	A	0.745807
***SEC22B***	NM_004892.5:p.Arg132*/c.394C>T	Cga/Tga	chr1	145112420	145112420	C	T	-
***ZNF117***	NM_015852.3:p.Arg428*/c.1282C>T	Cga/Tga	chr7	64438667	64438667	G	A	0.881789
***ANKDD1B***	NM_001276713.1:p.Trp480*/c.1439G>A	tGg/tAg	chr5	74965122	74965122	G	A	0.506989
***PRIM2***	NM_000947.4:p.Gln325*/c.973C>T	Cag/Tag	chr6	57398270	57398270	C	T	-
***OR51Q1***	NM_001004757.2:p.Arg236*/c.706C>T	Cga/Tga	chr11	5444136	5444136	C	T	0.453874
***MUC19***	NM_173600.2:p.Cys1238*/c.3714C>A	tgC/tgA	chr12	40834955	40834955	C	A	0.518371
***USP29***	NM_020903.2:p.Tyr913*/c.2739C>A	taC/taA	chr19	57642782	57642782	C	A	0.952077
***PRM3***	NM_021247.2:p.Arg104*/c.310C>T	Cga/Tga	chr16	11367143	11367143	G	A	1
***MAP3K14***	NM_003954.4:p.Ser902*/c.2705C>G	tCa/tGa	chr17	43342141	43342141	G	C	0.718251
***MS4A12***	NM_017716.2:p.Gln71*/c.211C>T	Caa/Taa	chr11	60265002	60265002	C	T	0.478235
***PKD1L3***	NM_181536.1:p.Arg789*/c.2365C>T	Cga/Tga	chr16	72001136	72001136	G	A	0.263379
***TPTE***	NM_199261.3:p.Arg229*/c.685C>T	Cga/Tga	chr21	10942756	10942756	G	A	-
***OR5AR1***	NM_001004730.1:p.Gln19*/c.55C>T	Cag/Tag	chr11	56431216	56431216	C	T	0.623003
***CENPM***	NM_001110215.1:p.Arg3*/c.7C>T	Cga/Tga	chr22	42336172	42336172	G	A	0.239217
***MAGEB16***	NM_001099921.1:p.Arg272*/c.814C>T	Cga/Tga	chrX	35821127	35821127	C	T	0.620397
***CLDN5***	NM_003277.3:p.Gln37*/c.109C>T	Cag/Tag	chr22	19511925	19511925	G	A	0.496805

* Frequency in 1000 Genomes Project.

**Table 3:** Significant pathways identified in the seven whole-genome samples of CRC patients.

Patient ID	Pathway ID	Pathway Description	FDR *	KEGG Pathway	FDR *
F1	GO.0004984	Olfactory receptor activity	0.0000169	Olfactory transduction	0.00000315
	GO.0004930	G-protein coupled receptor activity	0.000486		
	GO.0004888	Transmembrane signalling receptor activity	0.00497		
F2	GO.0004984	Olfactory receptor activity	0.00000228	Olfactory transduction	0.000000432
	GO.0004930	G-protein coupled receptor activity	0.0000529		
F5	GO.0004984	Olfactory receptor activity	0.00133	Olfactory transduction	0.000207
	GO.0004888	Transmembrane signalling receptor activity	0.00423		
	GO.0004930	G-protein coupled receptor activity	0.00602		
F8	GO.0004984	Olfactory receptor activity	0.00676	Olfactory transduction	0.00097
F12	GO.0004984	Olfactory receptor activity	0.000105	Olfactory transduction	0.0000179
	GO.0004930	G-protein coupled receptor activity	0.000229		
	GO.0004888	Transmembrane signalling receptor activity	0.000411		
F18	GO.0004984	Olfactory receptor activity	0.00064	Olfactory transduction	0.000101
	GO.0004930	G-protein coupled receptor activity	0.000768		
	GO.0004888	Transmembrane signalling receptor activity	0.00569		
F19	GO.0004984	Olfactory receptor activity	0.000548	Olfactory transduction	0.0000863
	GO.0004930	G-protein coupled receptor activity	0.00444		

* False discovery rate (FDR) ≤ 0.05.

**Table 4:** Insertions and deletions identified in the seven whole-genome samples of CRC patients.

Function	Gene	Transcript	Chr	Start	End	Ref	Obs
In-frame insertion	*CDK11B*	NM_001787.2:p.Arg127_Glu128insLysGluArg/c.379_380insAAGAAA	1	1647893	1647893	C	CTTTCTT
Frameshift variant	*CCDC144NL*	NM_001004306.1:p.Lys213fs/c.638delA	17	20768755	20768755	CT	C
Frameshift variant	*CCDC144NL*	NM_001004306.1:p.Lys211_Gly212fs/c.631_632insG	17	20768762	20768762	T	TC
In-frame insertion	*GOLGA8R*	NM_001282484.1:p.Gln271_Asp272insGlnGln/c.813_814insCAA	15	30700168	30700168	C	CTTG
Disruptive in-frame deletion	*MAFA*	NM_201589.3:p.His207_His208del/c.621_623delCCA	8	144511953	144511953	ATGG	A
Frameshift variant	*MUC6*	NM_005961.2:p.Pro1571fs/c.4712delC	11	1018088	1018088	TG	T
Frameshift variant	*MUC6*	NM_005961.2:p.Pro1569_Pro1570fs/c.4707_4708insA	11	1018093	1018093	G	GT
Frameshift variant	*PRIM2*	NM_000947.4:p.Glu297_Asn298fs/c.890_891insA	6	57398186	57398186	G	GA

## Data Availability

The data presented in this study are available on request from the corresponding author.

## Supplementary Materials

The following are available online at https://www.mdpi.com/article/10.3390/genes12091448/s1, Table S1: Alignment statistics of the whole genome probands, Table S2: Summary of SNVs in seven whole genome samples from CRC patients, Table S3: The most significant pathway enriched by genes harboured the nonsense mutation by Reactome tool in a) F1 b) F2 c) F5 d) F8 e) F12 f) F18 g) F19.
