# Supplementary material for: Whole-Genome Profiles of Malay Colorectal Cancer Patients with Intact MMR Proteins
Source: Genes (Basel). 2021 Sep 20;12(9):1448. doi: 10.3390/genes12091448 (PMC8471947; doi:10.3390/genes12091448)
Supplement: Supplementary file 1 [file genes-12-01448-s001.zip › Supp Table S1.pdf]

Supplementary Table S1: Alignment statistics of the whole genome probands.

Samples	F1	F2	F5	F8	F12	F18	F19
Raw reads	$1.21x10^{9}$	$1.23x10^{9}$	$1.15x10^{9}$	$1.08x10^{9}$	$1.25x10^{9}$	$1.26x10^{9}$	$1.15x10^{9}$
Clean reads	$1.20x10^{9}$	$1.22x10^{9}$	$1.14x10^{9}$	$1.00x10^{9}$	$1.24x10^{9}$	$1.25x10^{9}$	$1.14x10^{9}$
Clean bases (bp)	$1.88x10^{11}$	$1.83x10^{11}$	$1.71x10^{11}$	$1.51x10^{11}$	$1.86x10^{11}$	$1.87x10^{11}$	$1.71x10^{11}$
Mapped reads	$1.14x10^{9}$	$1.16x10^{9}$	$1.09x10^{9}$	$9.42x10^{9}$	$1.17x10^{9}$	$1.18x10^{9}$	$1.08x10^{9}$
Mapped bases (bp)	$1.71x10^{11}$	$1.74x10^{11}$	$1.63x10^{11}$	$1.41x10^{11}$	$1.76x10^{11}$	$1.77x10^{11}$	$1.62x10^{11}$
Mapping rate (%)	94.91	95.02	95.15	93.89	94.52	94.59	94.83
Unique reads	$1.11\ x10^{9}$	$1.13\ x10^{9}$	$1.06\ x10^{9}$	$9.20\ x10^{8}$	$1.15\ x10^{9}$	$1.15\ x10^{9}$	$1.05\ x10^{9}$
Unique bases (bp)	$1.67x10^{11}$	$1.70\ x10^{11}$	$1.59\ x10^{11}$	$1.38\ x10^{11}$	$1.72\ x10^{11}$	$1.73\ x10^{11}$	$1.58\ x10^{11}$
Unique rate (%)	97.63	97.63	97.65	97.61	97.58	97.57	97.50
Average sequencing depth (fold)	52.39	52.78	49.72	43.84	53.41	53.32	49.31
Coverage (%)	99.81	99.10	99.09	99.36	99.56	99.09	99.19
