# Supplementary material for: Whole-Genome Profiles of Malay Colorectal Cancer Patients with Intact MMR Proteins
Source: Genes (Basel). 2021 Sep 20;12(9):1448. doi: 10.3390/genes12091448 (PMC8471947; doi:10.3390/genes12091448)
Supplement: Supplementary file 1 [file genes-12-01448-s001.zip › Supp Table S2.pdf]

Supplementary Table S2: Summary of SNVs in seven whole genome samples from CRC patients.

Samples	F1	F2	F5	F8	F12	F18	F19
Genome	hg19	hg19	hg19	hg19	hg19	hg19	hg19
Total	3255512	3294111	3278396	3231785	3303831	3277862	3295827
1000genome and dbsnp141	3129832	3167302	3150526	3109874	3176391	3147136	3168400
1000genome specific	36019	36841	35779	33908	36104	39029	36055
dbSNP141 specific	49254	48773	49358	46943	48180	49567	49469
dbSNP rate	97.65%	97.63%	97.61%	97.68%	97.60%	97.52%	97.63%
Novel	40407	41195	42733	41060	43156	42130	41903
Hom	1368161	1335716	1365694	1348279	1197106	1334555	1331790
Het	1887351	1958395	1912702	1883506	2106725	1943307	1964037
Synonymous	10717	10610	10480	10503	10780	10585	10545
Missense	9601	9581	9516	9449	9620	9370	9500
Stopgain	81	73	82	73	78	73	73
Stoploss	39	33	32	31	29	31	34
Startgain	761	720	733	723	736	744	732
Startloss	18	17	26	27	25	20	19
Exonic	22374	22209	22038	22036	22487	22040	22147
Splicing	136	142	146	143	138	138	141
NcRNA	5870	6134	5931	5875	6177	6223	6007
UTR5	4175	4144	4165	4118	4144	4256	4175
UTR3	21078	21461	20977	20906	21465	21162	21418
Intronic	1284613	1288415	1289826	1273415	1302249	1286561	1293052
Upstream	44054	44392	43965	43185	44532	44161	43761
Downstream	42679	43315	43106	42308	43074	43111	42724
Intergenic	1830533	1863899	1848242	1819799	1859565	1850210	1862402
SIFT	1759	1762	1798	1767	1824	1711	1752
Ti/Tv	2.1322	2.1295	2.1299	2.1275	2.131	2.13	2.1263
dbSNP Ti/Tv	2.1343	2.1314	2.1315	2.1289	2.1332	2.1321	2.1285
Novel Ti/Tv	1.9249	1.9198	1.9447	1.9552	1.9418	1.9035	1.9449
