# Supplementary material for: Whole-Genome Profiles of Malay Colorectal Cancer Patients with Intact MMR Proteins
Source: Genes (Basel). 2021 Sep 20;12(9):1448. doi: 10.3390/genes12091448 (PMC8471947; doi:10.3390/genes12091448)
Supplement: Supplementary file 1 [file genes-12-01448-s001.zip › Supp Table S3.pdf]

Supplementary Table S3: The most significant pathway enriched by genes harboured the nonsense mutation by Reactome tool in a) F1 b) F2 c) F5 d) F8 e) F12 f) F18 g) F19

a)

Pathway name	Entities				Reactions	
	found	ratio	p-value	FDR*	found	ratio
Olfactory Signaling Pathway	12 / 432	0.031	6.13e-06	0.001	1 / 2	1.72e-04

b)

Pathway name	Entities				Reactions	
	found	ratio	p-value	FDR*	found	ratio
Olfactory Signaling Pathway	12 / 432	0.031	1.20e-06	1.91e-04	1 / 2	1.72e-04

c)

Pathway name	Entities				Reactions	
	found	ratio	p-value	FDR*	found	ratio
Olfactory Signaling Pathway	10 / 432	0.031	1.74e-04	0.042	1 / 2	1.72e-04

d)

Pathway name	Entities				Reactions	
	found	ratio	p-value	FDR*	found	ratio
Olfactory Signaling Pathway	9 / 432	0.031	3.59e-04	0.057	1 / 2	1.72e-04

e)

Pathway name	Entities				Reactions	
	found	ratio	p-value	FDR*	found	ratio
Olfactory Signaling Pathway	11 / 432	0.031	2.68e-05	0.006	1 / 2	1.72e-04

f)

Pathway name	Entities				Reactions	
	found	ratio	p-value	FDR*	found	ratio
Olfactory Signaling Pathway	10 / 432	0.031	6.98e-05	0.011	1 / 2	1.72e-04

g)

Pathway name	Entities				Reactions	
	found	ratio	p-value	FDR*	found	ratio
Olfactory Signaling Pathway	10 / 432	0.031	7.37e-05	0.011	1 / 2	1.70e-04

*False discovery rate (FDR)≤0.05; p-value<0.05
